# Forces of the Living Organism

**Published:** 1863-05

**Authors:** 


### FORCES OF THE LIVING ORGANISM.

In the course of an able address on physiology before the British Medical Association, Dr. W. Sharpley made (*Med. Times and Gazette*) the following comprehensive remarks upon this subject: "And now, gentlemen, in drawing to a close, I may be expected to say a word on the prevailing views as to the powers which animate the living organism.

"I have already remarked that many of the processes of the living economy issue in physical or chemical results, and I have stated that the more or less close relation subsisting between these results, so far as they can be estimated, and the consumption and oxidation of nutriment, as indicated in respiration and excretion, would seem to show that the chemical and mechanical forces developed are derived from an extrinsic source; but, at the same time, that there are energies displayed in the living body not yet estimated in amount, concerning which, therefore, there is not the same clear evidence. I refer especially to the nervous energy.

"In speaking, however, of the nerve force I understand that force which is common to all creatures possessing a nervous system, from the highest to the lowest. I do not refer to the highest attributes of man, to his sense of moral responsibility, his consciousness of dependence on a higher power, and his aspiration after perfection in a future state.

"This nervous force has long been likened to electricity; but rather through a vague perception of anology than from any rigorous comparison. It is true that electric force is developed in the nerves, and even exhibits modifications connected with different conditions of nervous action. Still, it must be borne in mind that the evolution of electricity is a common accompaniment of various processes, involving chemical change, whether within the body or in external nature; and the tendency of recest speculation is not toward identification of the nerve force with electricity, but rather to suggest that the two stand related in the same way as electricity and other physical forces are related to each other; that is, as manifestations of a common force or energy, of which they, severally, are the special modifications.

"Since the memorable experiments of Count Rumford on the heat of friction, which led that philosopher to the conclusion that heat is a form of motion, and the determination by Dr. Joule at a later period, of the equivalent of heat expressed in mechanical work, the doctrine of commutability and equivalence of force, first applied to these two agencies, has extended itself to the other forces operating in the material universe. Accordingly, the opinion is now gaining consistency and acceptance, that mechanical energy, heat, light, chemical action, electricity, and magnetism, are mutually convertible, and are respectively equivalent to each other; moreover, that they are probably all the expression of a common force which manifests itself under these several modifications, according to the different material or dynamic conditions in which it operates.

"Now, the belief has some time prevailed that the nervous, with perhaps other forms of organic energy, has its place in the same circle of reciprocally productive and equivalent forces; and not being electricity more than it is heat or chemical affinity, yet stands related to electricity and the other forces in the same way that they are related to each other.

"But supposing this probable doctrine to be proved and to betoken a signal advance in physiology, are we come to the end of our inquiries? are we thereby enabled to explain even the most characteristic phenomena of the living organism?

"By mechanical force duly applied, a fabric may be woven, as well, or perhaps better, than by human hands; but by what intelligent prearrangement is the pattern determined and finally brought out? So in the production and development of an animal, and in its subsequent workings,—given the force or forces operating,—how are the determinate forms and qualities of organism produced?

"To all our most exquisite means of scrutiny, the ovum, as it proceeds from the parent, presents nothing to indicate the course of its future development; and yet we speedily can discern in it the traces of the new being, and recognize the successive appearance of each new member and organ—in due time aud form and proportion—until the body is built up and completed after the pattern of the parent. We can perceive nothing in the ovum of man to distinguish it from that of a quadruped, although their final destination is so different. We are constrained, therefore, to admit some pre-existent condition, to us inscrutable, which determine the specific direction in which the forces, acting in development, although probably supplied from without, must operate within the organism. And the marvel reaches its height when we reflect, that not the character of the species merely, but the individual likeness of the parent—aye, of both parents—displays itself in the offspring; and not alone in bodily feature, but often also in intellectual and moral peculiarities.

"Then, not alone in regulated form and proportion, do the parts appear, but all fitting harmoniously the one to the other, and each in its appointed time. The periods of incubation and gestation, different but determinate in each species; the regulated time of consolidation and completion of the bones of the skeleton; of the eruption and succession of the teeth; the periods of maturity and decline of the whole body and of particular organs; and a host of examples supplied by the history of the lower member of the creation, serve to illustrate that conspicuous law of subordination to time in the phenomena of the organic world, which Mr. Paget has aptly designated as the 'chronometry of life."

"Now, while we can in many cases discern the purpose of these adaptations of form, proportion, and time, and perceive how they, as it were, fit in with, although not apparently produced by, the outward circumstances in which the organism is placed; and while we must revere the infinite wisdom by which they are harmoniously brought about, we are still utterly at a loss to explain them by reference to efficient causes. In some of the lowest tribes of animals, it is true, the results are affected more or less by physical influences, but these influences operate upon internal conditions, existing independently. In the human body, even, you may cramp the growth of a Chinese foot or flatten a Carib skull, but this is suppression or distortion, not formation.

"The growth of a finger or a tooth may be traced, and various steps in the process explained; but the acquirement by these and other parts, and indeed by the entire body, of their characteristic form and proportion, is still an inscrutable, at least an unpenetrated mystery. Unpenetrated, I mean, as regards the physical or efficient causes of the phenomena; for the purpose or final cause is often patent; and hence we see that teleogical explanation holds, and doubtless must continue to hold, a large place in physiology.

"But, finally, shall we, on that account, censure as rash or stigmatize as impious all attempts to go farther? Shall we presumptuously set limits to the scope of those inquiring faculties which God has conferred on man, or prejudge and reject by anticipation, conclusions to which their rational and reverential exercise may lead? Assuredly not. Let us not, therefore, with narrow views of the scheme of Providence, worthy of a darker age, join in blindly denouncing the genial effort of one of the foremost men of science in our time, to refer mutations of organic form and the origin of species to natural causes of known operation. Faint as some may deem the prospect of success of Mr. Darwin's great attempt, let none condemn its tendency. Should it ever be shown that the wonderful adaptation and harmonious working, so conspicuous in the living creation, have been brought about by the operation of great natural causes, originally ordained by the Author of the universe, and acting through countless ages of time, surely such an issue could but tend to enlighten and exalt our conceptions of creative wisdom.—*Drug. Circ.*

